# Childhood adversity moderates the association between serum cortisol and earlier-onset posttraumatic stress disorder

**DOI:** 10.1186/s13034-026-01129-x

**Published:** 2026-06-29

**Authors:** Jae-Min Kim, Hee-Ju Kang, Ju-Wan Kim, Ju-Yeon Lee, Hyunseok Jang, Jung-Chul Kim, Sung-Wan Kim, Il-Seon Shin

**Affiliations:** 1 Department of Psychiatry, Chonnam National University Medical School, 160 Baekseoro, 12 Dong-gu, Gwangju, 61469 Republic of Korea; 2 Division of Trauma, Department of Surgery, Chonnam National University Medical School and Hospital, Gwangju, Republic of Korea

**Keywords:** Cortisol, Childhood adversity, Posttraumatic stress disorder, Moderating effect, Earlier-onset

## Abstract

**Background:**

The role of cortisol in Posttraumatic stress disorder (PTSD) remains controversial due to inconsistent findings. Childhood adversity may recalibrate the hypothalamic–pituitary–adrenal (HPA) axis, altering the prognostic value of cortisol. Furthermore, the biological mechanisms underlying earlier- versus delayed-onset PTSD trajectories may differ.

**Methods:**

A total of 895 patients hospitalized for physical injuries were prospectively followed for 2 years. Baseline serum cortisol levels and childhood adversity were assessed within one month of injury. PTSD diagnoses and onset timing were determined at 3, 6, 12, and 24 months using the Clinician-Administered PTSD Scale for DSM-5. Logistic regression models evaluated individual and interactive effects of cortisol and childhood adversity on PTSD onset, adjusting for relevant covariates.

**Results:**

Neither baseline serum cortisol levels nor childhood adversity independently predicted PTSD onset. However, a significant interaction was observed for earlier-onset PTSD, such that lower cortisol levels were associated with increased risk only among individuals without childhood adversity. This interaction was not observed for delayed-onset PTSD. Cortisol levels were not correlated with childhood adversity.

**Conclusion:**

Baseline cortisol predicts earlier-onset PTSD only in the absence of childhood adversity, suggesting that developmental context modifies the prognostic significance of cortisol. Biological risk stratification for PTSD must consider developmental history and symptom onset timing.

**Supplementary Information:**

The online version contains supplementary material available at https://doi.org/10.1186/s13034-026-01129-x.

## Background

Posttraumatic stress disorder (PTSD) is a debilitating condition characterized by persistent intrusive symptoms, avoidance, and hyperarousal following trauma exposure. Identifying early biological markers that predict PTSD development is essential for targeted prevention [1]. Among various candidates, cortisol—the primary glucocorticoid effector of the hypothalamic–pituitary–adrenal (HPA) axis—has been a focal point due to its role in regulating the neuroendocrine response to stress, fear extinction, and memory consolidation [2].

Cortisol facilitates adaptive responses to acute stress by mobilizing energy resources, regulating inflammatory processes, and modulating fear learning and memory consolidation. The “hypocortisolism hypothesis” of PTSD proposes that insufficient cortisol activity around the time of trauma may impair stress containment and thereby increase vulnerability to PTSD. However, empirical support for this hypothesis has been mixed. Individual studies have reported elevated, reduced, or unchanged cortisol levels among individuals who subsequently develop PTSD [3–5], and meta-analytic findings have also varied according to cortisol specimen, sampling timing, trauma characteristics, and study design [6–8]. For example, meta-analytic evidence on urinary cortisol has supported lower cortisol levels in PTSD [7], whereas studies of cortisol reactivity to trauma-related stimuli have shown more heterogeneous associations with later PTSD symptoms [8]. These inconsistencies indicate that cortisol-related vulnerability is unlikely to operate uniformly across all trauma-exposed individuals. Rather, the prognostic significance of cortisol may depend on contextual and developmental moderators, including early-life adversity, post-trauma biological adaptation, and the temporal trajectory of PTSD symptom emergence.

Childhood adversity is a potent determinant in the functional programming of the HPA axis [9]. Early-life stress is known to induce enduring alterations in glucocorticoid receptor sensitivity and neurobiological feedback mechanisms, resulting in a persistent recalibration of the stress-response system [10]. Consequently, an identical cortisol concentration observed in adulthood may represent fundamentally different physiological or adaptive states depending on an individual’s developmental history of maltreatment [11]. Such developmental “biological embedding” suggests that the relationship between acute cortisol levels and the risk of developing PTSD—whether manifest as earlier or delayed onset—may be conditional upon the presence of early-life adversity.

Furthermore, the timing of PTSD onset— specifically the distinction between earlier-onset and delayed-onset PTSD—may be important for understanding heterogeneity in post-trauma adaptation, but it is often overlooked in biomarker research. In the Diagnostic and Statistical Manual of Mental Disorders, Fifth Edition (DSM-5), the delayed expression specifier is applied when full diagnostic criteria are not met until at least six months after the traumatic event [12]. Consistent with this definition, prospective studies and systematic reviews have commonly distinguished PTSD emerging within the first several months after trauma from delayed-onset or delayed-expression PTSD [13, 14]. Earlier-onset PTSD may be more closely related to acute post-trauma processes, including early stress-system dysregulation, fear learning, and initial symptom consolidation. In contrast, delayed-onset PTSD may involve mechanisms that unfold over time, such as cumulative psychosocial stress, subsequent trauma exposure, sensitization, kindling, or progressive neurobiological adaptation [13–15]. These observations suggest that biological predictors measured soon after trauma may not operate uniformly across earlier- and delayed-onset PTSD trajectories, but this possibility remains insufficiently examined for HPA-axis markers such as cortisol.

Therefore, this study aims to investigate whether childhood adversity moderates the association between baseline serum cortisol and the development of earlier- versus delayed-onset PTSD. We hypothesized that cortisol’s prognostic significance would be conditional on childhood adversity exposure and would differ across PTSD onset trajectories.

## Methods

### Study design and participants

This study was conducted as part of the Biomarker-based Diagnostic Algorithm for Post-Traumatic Syndrome (BioPTS) project, a large-scale prospective longitudinal cohort study designed to identify biological and psychosocial predictors of PTSD [16]. The present report is a secondary analysis of the BioPTS cohort. Baseline cortisol, childhood adversity, and repeated PTSD diagnostic assessments were included prospectively in the parent protocol, whereas the current analytic classifications—earlier- versus delayed-onset PTSD, any versus no childhood adversity, and higher versus lower cortisol levels—were operationalized for the present research question and are described below.

Participants were consecutively recruited from adults hospitalized for physical injuries at the Trauma Center of Chonnam National University Hospital (Gwangju, South Korea) between June 2015 and January 2021. Eligible individuals (see Supplementary Material) underwent baseline psychiatric assessments within one month of hospitalization. PTSD outcomes were evaluated at 3, 6, 12, and 24 months after the index trauma. The study protocol was approved by the Institutional Review Board of Chonnam National University Hospital (CNUH-2015-148), and all participants provided written informed consent in accordance with the Declaration of Helsinki.

### Baseline assessments

#### Serum cortisol

To minimize the impact of circadian rhythm and acute stress, venous blood samples were collected between 08:00 and 09:00 AM after an overnight fast and a 30-minute resting period. Serum cortisol concentrations were determined using a competitive immunoassay (Elecsys Cortisol II; Roche Diagnostics, Mannheim, Germany) at a certified central laboratory. For analysis, cortisol was examined both as a continuous variable and as a dichotomized variable. Because no established clinical cutoff exists for serum cortisol in predicting post-trauma PTSD, the dichotomous classification was based on the sample median (14.3 µg/dL), which provided balanced groups for stratified presentation and facilitated interpretation of the interaction analyses. To minimize reliance on this data-driven threshold and preserve information from the original measurement scale, cortisol was also modeled continuously [17].

#### Childhood adversity

The Adverse Childhood Experiences (ACE) questionnaire [18] was used to assess exposure to trauma prior to age 18. This 10-item instrument covers categories of abuse (physical, emotional, sexual), neglect (physical, emotional), and household dysfunction (e.g., domestic violence, parental divorce, or substance abuse). Total ACE scores (range 0–10) were examined continuously and categorically. Consistent with prior literature, childhood adversity was operationalized dichotomously as the presence of at least one ACE (score ≥ 1) versus none (score = 0) [18, 19].

#### Covariates

Potential confounders were adjusted for across four primary domains.

1. Demographic profiles

At the outset of the study, we documented several socio-demographic variables to serve as baseline covariates. These included chronological age, gender, and the total years of formal education. Additionally, we recorded each participant’s domestic living arrangements (living alone versus with others) and current vocational status (employed versus unemployed).

2) Pre-existing clinical and psychosocial factor

Participants’ psychiatric history was screened for prior diagnoses of depressive disorders, panic disorder, agoraphobia, social phobia, and generalized anxiety disorder. Lifetime exposure to traumatic events was quantified using the Life Experiences Survey [20], with participants categorized according to the presence or absence of any prior trauma. General medical health was represented by the total number of chronic physical conditions across 15 systemic categories. Lifestyle-related factors, specifically tobacco use (current smoker vs. non-smoker) and alcohol consumption patterns—the latter assessed via the Alcohol Use Disorders Identification Test (AUDIT) [21]—were also included. Physiological data included Body Mass Index (BMI) calculations. Furthermore, we evaluated baseline psychological resources, namely resilience and perceived social support, using the Connor-Davidson Resilience Scale (CD-RISC) [22] and the Multidimensional Scale of Perceived Social Support (MSPSS) [23]. In these assessments, lower scores on the CD-RISC and MSPSS were interpreted as indicators of higher psychological vulnerability.

3) Characteristics of the index trauma

To evaluate the clinical impact of the traumatic event, we utilized the Injury Severity Score (ISS) [24] and Glasgow Coma Scale (GCS) [25] as described in the Eligibility criteria, in the Supplementary Material; higher ISS scores and lower GCS scores served as indicators of more pronounced injury-related symptomatology. Furthermore, in line with previous findings that intentionality significantly influences PTSD development [26], the nature of the trauma was classified as either intentional (e.g., interpersonal violence) or unintentional (e.g., accidental injury) based on the Life Events Checklist (LEC-5) [27].

4)  Peri-traumatic psychological and physiological status

Participants’ psychological and functional status during the peri-traumatic period—defined as the interval from the index injury to the baseline evaluation—was assessed. Acute depressive and anxiety symptoms were measured using the Hospital Anxiety and Depression Scale (HADS) depression (HADS-D) and anxiety (HADS-A) subscales [28], with higher scores indicating more severe symptoms. Additionally, physiological status was monitored by recording the resting heart rate at the time of the baseline evaluation.

### PTSD assessment and onset classification

PTSD diagnoses were established using the Clinician-Administered PTSD Scale for DSM-5 (CAPS-5) [29], which was administered at each follow-up assessment. Diagnostic status was determined in accordance with DSM-5 criteria, requiring the fulfillment of specific symptom clusters and the presence of clinically significant functional impairment [12]. Participants were further classified based on the timing of symptom emergence relative to the index trauma. Earlier-onset PTSD was defined as the manifestation of symptoms within the initial six months post-trauma. In contrast, delayed-onset PTSD was defined as symptom onset occurring at six months or later, a classification consistent with the DSM-5 ‘with delayed expression’ specifier.

### Statistical analysis

To ensure the accurate categorization of PTSD onset trajectories, participants who discontinued follow-up prior to the six-month assessment were excluded from the final analytic sample. Baseline demographic and clinical characteristics were compared across groups using independent-sample t-tests for continuous variables and chi-square (χ²) tests for categorical data. The bivariate association between baseline serum cortisol levels and ACE scores was evaluated using Spearman’s rank correlation coefficient (*ρ*).

The primary analysis involved multivariable logistic regression to investigate the independent and interactive contributions of cortisol and ACE to PTSD onset trajectories. To examine potential moderation, a cross-product interaction term (cortisol × early adversity) was incorporated into the regression models. Consistent with our a priori hypothesis, these analyses were conducted separately for earlier-onset and delayed-onset PTSD to identify trajectory-specific predictors. To further confirm the robustness of the interaction, additional models were tested using both cortisol and ACE as continuous variables.

Variables significantly associated with early adversity, cortisol levels, or PTSD development (*P* < 0.05) in univariate analyses, as well as clinically relevant factors (e.g., age, sex, and injury severity), were included as covariates in the adjusted models. Multicollinearity was monitored using the Variance Inflation Factor (VIF), with values below 10 indicating acceptable model stability. All statistical tests were two-tailed, and significance was defined as *P* < 0.05. Data analyses were performed using IBM SPSS Statistics for Windows, version 21.0 (IBM Corp., Armonk, NY, USA).

## Results

### Participant characteristics and PTSD trajectories

A total of 1,002 eligible individuals were initially enrolled and provided baseline blood samples. Of these, 107 participants (10.7%) withdrew before the six-month follow-up and were consequently excluded, resulting in a final analytic sample of 895 individuals (Fig. S1). Attrition analysis indicated no significant differences in baseline demographic or clinical characteristics between participants who completed the study and those who were lost to follow-up (all *P* > 0.10).

Over the two-year prospective follow-up period, 107 participants (11.9%) received a PTSD diagnosis. This group included 76 cases of earlier-onset PTSD (8.4%) and 31 cases of delayed-onset PTSD (3.5%). Compared to the non-PTSD group, individuals who developed PTSD were significantly younger, more likely to be female, and reported higher educational attainment. Furthermore, these participants exhibited higher rates of pre-existing psychiatric disorders and cumulative lifetime trauma exposure, as well as more severe baseline anxiety and depressive symptoms (Table S1).

Analysis of the primary independent variables revealed that childhood adversity was significantly associated with lower levels of resilience, intentional nature of the index injury, and elevated depressive symptomatology (Table 1). Conversely, baseline serum cortisol levels demonstrated no significant associations with any sociodemographic or clinical parameters (Table 2). Based on these univariate findings and considering multicollinearity, eight variables were selected as covariates for the subsequent multivariable analyses: age, sex, educational level, prior psychiatric history, cumulative lifetime trauma, CD-RISC scores, the nature of the index injury (intentional vs. unintentional) and HADS-D scores.

**Table 1:** Baseline characteristics according to childhood adversity (CA) status

	Absent CA (*N* = 745)	Present CA (*N* = 150)	Statistical coefficients	*P*-value^a^
Age, mean (SD) years	56.5 (16.7)	54.4 (18.6)	t = + 1.315	0.190
Sex, N (%) female	227 (30.5)	45 (30.0)	χ^2^ = 0.013	0.909
Education, mean (SD) years	10.8 (4.1)	10.7 (4.0)	t = + 0.127	0.899
Marital status, N (%) unmarried	243 (32.6)	60 (40.0)	χ^2^ = 3.039	0.081
Living alone, N (%)	110 (14.8)	24 (16.0)	χ^2^ = 0.150	0.699
Unemployed status, N (%)	131 (17.6)	20 (13.3)	χ^2^ = 1.608	0.205
Previous psychiatric disorders, N (%)	46 (6.2)	15 (10.0)	χ^2^ = 2.877	0.090
Previous traumatic events, N (%)	31 (4.2)	8 (5.3)	χ^2^ = 0.412	0.521
Physical disorders, mean (SD) numbers	1.9 (1.9)	2.0 (2.3)	t=-0.589	0.557
Current smoker, N (%)	210 (28.2)	47 (31.3)	χ^2^ = 0.603	0.437
Alcohol Use Disorders Identification Test, mean (SD) scores	10.6 (10.1)	10.0 (9.6)	t = + 0.646	0.518
Body mass index, mean (SD)	23.5 (3.3)	24.1 (4.2)	t=-1.600	0.111
Connor-Davidson Resilience Scale, mean (SD) scores	68.7 (15.2)	64.8 (15.6)	t=-2.853	**0.004**
Multidimensional Scale of Perceived Social Support, mean (SD) scores	35.0 (10.0)	35.1 (10.3)	t=-0.135	0.893
Injury Severity Score, mean (SD) scores	14.8 (5.9)	14.6 (5.5)	t = + 0.360	0.719
Glasgow Coma Scale, mean (SD) scores	14.8 (0.7)	14.9 (0.6)	t=-0.330	0.741
Injury type, N (%) intentional	61 (8.2)	20 (13.3)	χ^2^ = 4.016	**0.045**
Hospital Anxiety Depression scale-anxiety subscale, mean (SD) scores	3.1 (3.5)	3.2 (3.8)	t=-0.375	0.708
Hospital Anxiety Depression scale-depression subscale, mean (SD) scores	4.7 (4.4)	5.7 (5.0)	t = + 2.378	**0.018**
Heart rate per minute, mean (SD)	78.6 (11.2)	79.2 (11.5)	t=-0.599	0.549

^a^t-tests or χ^2^ tests, as appropriate

Bold style indicates statistical significance (P-value < 0.05)

**Table 2:** Baseline characteristics according to baseline serum cortisol levels (higher: >14.3 vs. lower ≤ 14.3 µg/dL)

	Higher cortisol (*N* = 463)		Lower cortisol (*N* = 432)	Statistical coefficients	*P*-value^a^
Age, mean (SD) years	56.5 (16.3)		55.8 (17.8)	t = + 0.638	0.524
Sex, N (%) female	132 (28.5)		140 (32.4)	χ^2^ = 1.605	0.205
Education, mean (SD) years	10.7 (4.0)		10.8 (4.2)	t=-0.251	0.802
Marital status, N (%) unmarried	155 (33.5)		148 (34.3)	χ^2^ = 0.061	0.805
Living alone, N (%)	74 (16.0)		60 (13.9)	χ^2^ = 0.770	0.380
Unemployed status, N (%)	75 (16.2)		76 (17.6)	χ^2^ = 0.310	0.578
Previous psychiatric disorders, N (%)	25 (5.4)		36 (8.3)	χ^2^ = 3.029	0.082
Previous traumatic events, N (%)	23 (5.0)		16 (3.7)	χ^2^ = 0.857	0.355
Physical disorders, mean (SD) numbers	1.8 (2.0)	1.9 (2.0)		t=-0.937	0.349
Current smoker, N (%)	132 (28.5)	125 (28.9)		χ^2^ = 0.020	0.888
Alcohol Use Disorders Identification Test, mean (SD) scores	10.6 (10.0)	10.3 (10.0)		t = + 0.485	0.627
Body mass index, mean (SD)	23.5 (3.3)	23.7 (3.6)		t=-0.750	0.454
Connor-Davidson Resilience Scale, mean (SD) scores	65.2 (15.7)		65.6 (15.5)	t=-0.422	0.673
Multidimensional Scale of Perceived Social Support, mean (SD) scores	34.5 (10.1)		35.5 (10.1)	t=-1.447	0.148
Injury Severity Score, mean (SD) scores	15.0 (5.9)		14.4 (5.8)	t = + 1.527	0.127
Glasgow Coma Scale, mean (SD) scores	14.8 (0.8)		14.8 (0.7)	t=-0.005	0.996
Injury type, N (%) intentional	44 (9.5)	37 (8.6)		χ^2^ = 0.239	0.625
Hospital Anxiety Depression scale-anxiety subscale, mean (SD) scores	3.2 (3.6)	3.0 (3.5)		t = + 0.893	0.372
Hospital Anxiety Depression scale-depression subscale, mean (SD) scores	5.7 (5.0)	5.5 (4.8)		t = + 0.669	0.504
Heart rate per minute, mean (SD)	79.3 (11.5)	78.1 (10.9)		t = + 1.577	0.115

^a^ t-tests or χ^2^ tests, as appropriate

### Main effects of cortisol and childhood adversity

The mean baseline serum cortisol level for the total sample was 14.4 µg/dL (SD = 5.3), and childhood adversity was reported by 150 participants (16.8%). Spearman’s rank correlation coefficients showed no significant relationship between baseline cortisol levels and ACE scores (ρ = −0.021, *P* = 0.515). Furthermore, multivariable logistic regression analyses demonstrated that neither serum cortisol nor childhood adversity served as independent predictors of PTSD onset. This lack of a main effect was consistent for both earlier- and delayed-onset PTSD trajectories, persisting both before and after adjustment for pre-selected covariates (Table 3).

**Table 3:** Individual associations of baseline serum cortisol levels and childhood adversity (CA) with earlier- and delayed-onset posttraumatic stress disorder (PTSD) over 2 years

PTSD onset	Exposure	Group	*N*	No. (%) PTSD	Odds ratio (95% confidence interval)	
					Unadjusted	Adjusted^a^
Earlier	Cortisol	Higher (> 14.3 µg/dL)	452	33 (7.3)	Ref	Ref
		Lower (≤ 14.3 µg/dL)	412	43 (10.4)	1.48 (0.92–2.38)	1.69 (0.99–2.88)
	CA	Absent	723	65 (9.0)	Ref	Ref
		Present	141	11 (7.8)	0.86 (0.44–1.67)	0.80 (0.38–1.71)
Delayed	Cortisol	Higher (> 14.3 µg/dL)	430	11 (2.6)	Ref	Ref
		Lower (≤ 14.3 µg/dL)	389	20 (5.1)	2.07 (0.98–4.37)	2.16 (0.99–4.50)
	CA	Absent	680	22 (3.2)	Ref	Ref
		Present	139	9 (6.5)	2.07 (0.93–4.60)	2.23 (0.98–5.07)

Adjusted for age, sex, education, history of psychiatric disorders, previous traumatic events, resilience levels, injury type, and depression levels

### Interactive effects on PTSD onset

**Fig. 1:**
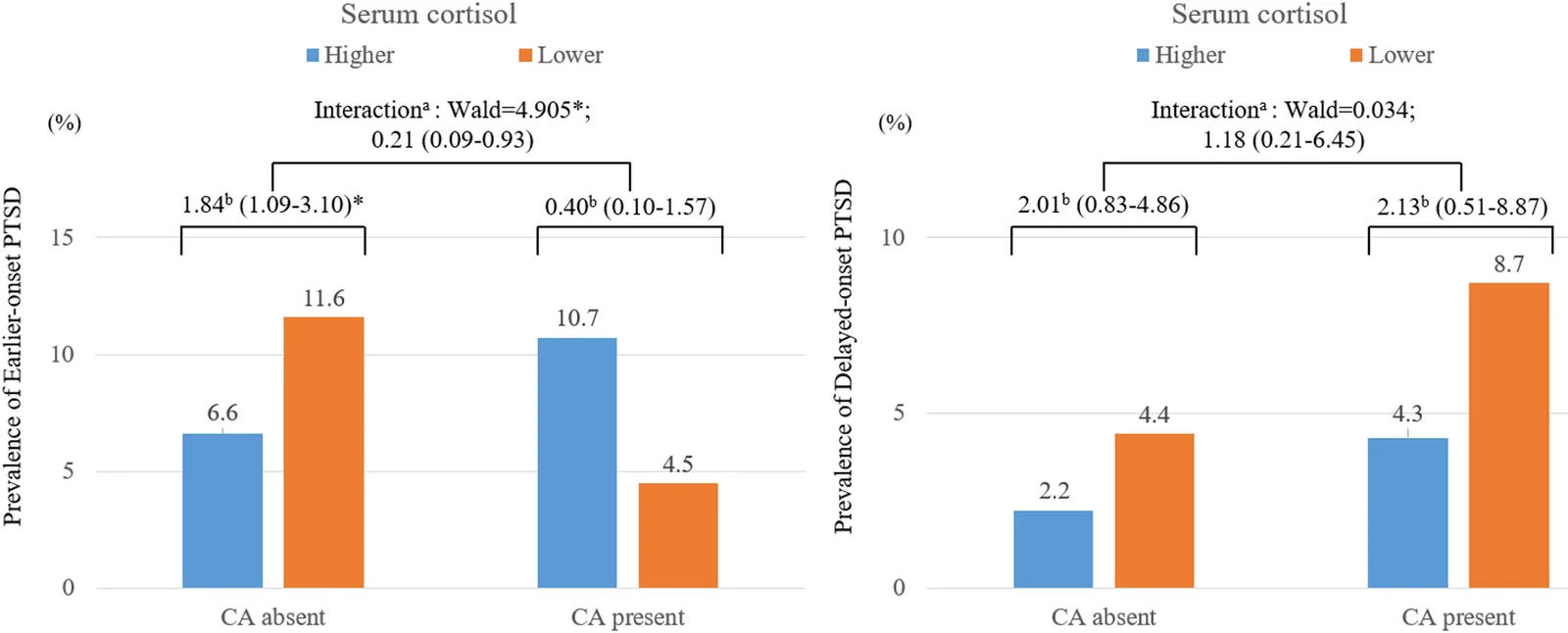
Childhood adversity moderates the association between baseline serum cortisol levels and earlier- but not delayed-onset posttraumatic stress disorder (PTSD) over 2 years. ^a^Interactive effects of childhood adversity (CA) on the association between baseline serum cortisol levels and the risk of PTSD onset were examined using logistic regression models; and ^b^odds ratios (ORs) and 95% confidence intervals (CIs) for earlier- and delayed-onset PTSD are shown for lower (≤ 14.3 µg/dL) versus higher (> 14.3 µg/dL) baseline serum cortisol levels, stratified by CA status. Models were adjusted for age, sex, education, history of psychiatric disorders, previous traumatic events, resilience, injury type, and depressive symptoms. ^*^P < 0.05

A significant interaction between serum cortisol levels and childhood adversity was identified specifically in relation to earlier-onset PTSD (Wald = 4.905, *P* = 0.018; Fig. 1). Among participants without a history of childhood adversity, lower cortisol levels were significantly associated with an increased risk of earlier-onset PTSD following covariate adjustment. In contrast, no such association between cortisol and PTSD risk was observed among individuals who had experienced childhood adversity.

Regarding delayed-onset PTSD, no significant interaction or main effects were detected (Wald = 0.034, *P* = 0.927; Fig. 1). These interactive patterns remained robust when both cortisol levels and ACE scores were modeled as continuous variables; specifically, the interaction term remained significant for earlier-onset PTSD (Wald = 5.121, *P* = 0.015) but did not reach significance for the delayed-onset trajectory (Wald = 0.306, *P* = 0.639).

## Discussion

The present prospective cohort study of trauma survivors hospitalized for physical injuries demonstrated that while baseline serum cortisol and childhood adversity did not independently predict PTSD development, their interaction significantly influenced the risk of earlier-onset PTSD. Specifically, lower cortisol levels were associated with an increased risk of PTSD onset within the first six months only among individuals without a history of childhood adversity. Notably, this interactive effect was absent for the delayed-onset trajectory, suggesting that the biological pathways to PTSD may differ depending on the timing of symptom emergence and developmental history.

### Cortisol, childhood adversity, and earlier-onset PTSD

The finding that lower cortisol predicts earlier-onset PTSD exclusively in participants without childhood adversity may help partly explain a potential explanation for the heterogeneous cortisol–PTSD associations reported in previous literature [6]. Cortisol is essential for stress containment, as it constrains inflammatory responses, facilitates fear extinction, and modulates memory consolidation. In individuals without prior childhood adversity, lower baseline cortisol may reflect a diminished capacity to mount an adequate neuroendocrine response to acute trauma, thereby increasing vulnerability to rapid symptom manifestation [30]. However, because cortisol was assessed only once, this interpretation should be regarded as provisional and requires confirmation using repeated or dynamic assessments of HPA-axis function.

In contrast, among those with childhood adversity, cortisol levels did not serve as a sensitive marker for PTSD risk. Early-life stress is known to induce long-term recalibration of the HPA axis through alterations in glucocorticoid signaling, receptor sensitivity, and feedback regulation [9, 31]. Consequently, similar cortisol concentrations in adulthood may represent biologically distinct stress-regulatory states depending on an individual’s developmental exposure [32]. The absence of a direct correlation between cortisol levels and ACE scores in our sample further supports the hypothesis that childhood adversity modifies the functional significance of cortisol rather than simply altering its absolute concentration.

### Differential mechanism of symptom timing

The significant cortisol × childhood adversity interaction observed for earlier-onset—but not delayed-onset—PTSD underscores the necessity of considering temporal trajectories when evaluating biological risk markers. Earlier-onset PTSD is likely driven by immediate failures in neurobiological stress regulation and fear processing following trauma exposure. In this acute window, cortisol may exert a time-sensitive protective role, particularly in individuals whose stress-response systems have not been previously disrupted by developmental adversity.

Conversely, delayed-onset PTSD may emerge through distinct mechanisms, such as the accumulation of psychosocial stressors, subsequent trauma, or progressive neurobiological changes that unfold over an extended period [13, 14]. These processes may depend less on acute HPA-axis function and more on downstream pathways, including chronic inflammatory, cognitive, or psychosocial factors [33]. Our results are consistent with, but do not prove, the hypothesis that early and delayed symptom trajectories are underpinned by diverging biological and environmental precursors.

### Strength and limitations

Despite its constraints, this study possesses several significant strengths that enhance the robustness of our findings. First, the prospective longitudinal design, characterized by repeated diagnostic evaluations, enabled a rigorous and clear differentiation between earlier-onset and delayed-onset PTSD trajectories. This temporal precision is critical for identifying trajectory-specific biomarkers that might otherwise be obscured in cross-sectional assessments. Second, by examining interaction effects in addition to main effects, our analysis provides a context-specific perspective on the heterogeneous findings reported in previous literature regarding cortisol and PTSD risk. This approach acknowledges the biological complexity of stress response systems. Most importantly, by synthesizing developmental history (childhood adversity) with temporal patterns of symptom emergence, this study demonstrates that biological markers do not function in isolation; rather, they may confer vulnerability only under specific developmental and temporal conditions. This integration provides a more nuanced framework for understanding the neurobiological underpinnings of PTSD.

However, certain limitations warrant consideration. First, serum cortisol was measured at a single time point, precluding the assessment of diurnal rhythms intra-individual variability, and cortisol reactivity to acute or repeated stress. Given the context-dependent nature of cortisol secretion, a single basal measurement may not adequately capture stable HPA-axis function [34]. Future studies incorporating repeated sampling, diurnal profiles, and stress-challenge paradigms are therefore needed to clarify the role of cortisol in PTSD onset. Second, childhood adversity was assessed retrospectively, which may be subject to recall bias, although the consistency between our continuous and categorical models suggests the findings are robust. Third, while we controlled for multiple pre-selected covariates, the possibility of residual confounding cannot be entirely excluded. Fourth, the relatively smaller number of delayed-onset cases might have limited the statistical power to detect more subtle effects within this specific subgroup. Finally, because the cohort consisted of patients hospitalized for physical injuries, the generalizability of these results to other trauma-exposed populations should be approached with caution.

## Conclusion

In conclusion, baseline serum cortisol levels predict earlier-onset PTSD only among individuals without a history of childhood adversity, while no such association is evident for the delayed-onset trajectory. These findings may partly account for inconsistencies in previous cortisol–PTSD research by suggesting that developmental history and symptom timing modify the prognostic significance of cortisol. However, given the use of a single cortisol measurement, clinical application of cortisol-based risk stratification remains premature. Future research should incorporate repeated biological measurements, multimodal biomarkers, and extended follow-up periods to further delineate the trajectory-specific mechanisms underlying the development of PTSD.

## Supplementary Information

Below is the link to the electronic supplementary material.

Supplementary Material 1.

## Data Availability

The data that support the findings of study are available from the corresponding author (J-M Kim) upon reasonable request.
