# Supplementary material for: Childhood adversity moderates the association between serum cortisol and earlier-onset posttraumatic stress disorder
Source: Child Adolesc Psychiatry Ment Health. 2026 Jun 29;20:129. doi: 10.1186/s13034-026-01129-x (PMC13591737; doi:10.1186/s13034-026-01129-x)
Supplement: Supplementary file 1 [file 13034_2026_1129_MOESM1_ESM.docx]

**Childhood adversity moderates the association between serum cortisol and earlier-onset posttraumatic stress disorder**

Jae-Min Kim^a*1^, Hee-Ju Kang^a1^, Ju-Wan Kim^a^, Ju-Yeon Lee^a^, Hyunseok Jang,^b^ Jung-Chul Kim,^b^ Sung-Wan Kim^a^, Il-Seon Shin^a^

Supplementary materials

**1. Eligibility criteria**

**2. References**

**Figure S1.** Participant flow and prevalence of posttraumatic stress disorder over 2 years following physical injury.

**Table S1.** Baseline characteristics according to post-traumatic stress disorder (PTSD) over 2-years.

**Eligibility criteria**

*1-1. Inclusion criteria*

i) individuals aged 18 years or older at the index injury.
ii) patients hospitalised for more than 24 hours after sustaining a moderate to severe physical injury measured by the Injury Severity Score (ISS) ≥9 [1].
iii) individuals sufficiently proficient in the Korean language to comprehend the study protocol.

*1-2. Exclusion criteria*

i) moderate or severe brain injury measured by the Glasgow Coma Scale (GCS) <10 [2].
ii) physical injuries resulting from suicide attempts.
iii) conditions hindering comprehensive psychiatric evaluation due to severe physical ailments.
iv) prior history of psychiatric disorders including psychotic disorder, bipolar disorder, or alcohol or substance use disorders other than depressive and anxiety disorders.
v) significant cognitive impairments due to organic mental or neurocognitive disorders.
vi) pre-existing convulsive disorders or a history of anticonvulsant use.

**References**

1. Baker SP, O’Neil B, Haddon W, et al (1974) The injury severity score: a method for describing patients with multiple injuries and evaluating emergency care. J Trauma 14:187–196. <https://doi.org/10.1097/00005373-197403000-00001>

2. Teasdale G, Jennett B (1974) Assessment of coma and impaired consciousness. A practical scale. Lancet 2:81–84. https://doi.org/10.1016/S0140-6736(74)91639-0

**Fig. S1.** Participant flow and prevalence of posttraumatic stress disorder over 2 years following physical injury.

| **Table S1**. Baseline characteristics according to post-traumatic stress disorder (PTSD) over 2-years. | | | | |
| --- | --- | --- | --- | --- |
|  | Absent  PTSD (N=788) | Present PTSD  (N=107) | Statistical coefficients | P-value^a^ |
|  |  |  |  |  |
| Age, mean (SD) years | 56.7 (17.2) | 52.2 (14.7) | t=+2.949 | **0.004** |
| Sex, N (%) female | 228 (28.9) | 44 (41.1) | χ^2^=6.615 | **0.010** |
| Education, mean (SD) years | 10.6 (4.1) | 11.8 (3.5) | t=-3.166 | **0.002** |
| Marital status, N (%) unmarried | 273 (34.6) | 30 (28.0) | χ^2^=1.837 | 0.175 |
| Living alone, N (%) | 124 (15.7) | 10 (9.3) | χ^2^=3.022 | 0.082 |
| Unemployed status, N (%) | 134 (17.0) | 17 (15.9) | χ^2^=0.084 | 0.772 |
| Previous psychiatric disorders, N (%) | 44 (5.6) | 17 (15.9) | χ^2^=15.749 | **<0.001** |
| Previous traumatic events, N (%) | 25 (3.2) | 14 (13.1) | χ^2^=22.206 | **<0.001** |
| Physical disorders, mean (SD) numbers | 1.9 (2.0) | 1.6 (1.8) | t=+1.421 | 0.156 |
| Current smoker, N (%) | 220 (27.9) | 37 (34.6) | χ^2^=2.042 | 0.153 |
| Alcohol Use Disorders Identification Test, mean (SD) scores | 10.5 (10.0) | 10.0 (10.2) | t=+0.537 | 0.592 |
| Body mass index, mean (SD) | 23.6 (3.5) | 23.7 (3.4) | t=-0.272 | 0.786 |
| Connor-Davidson Resilience Scale, mean (SD) scores | 65.4 (15.4) | 65.3 (17.4) | t=+0.099 | 0.921 |
| Multidimensional Scale of Perceived Social Support, mean (SD) scores | 34.8 (10.0) | 35.9 (10.8) | t=-1.052 | 0.293 |
| Injury Severity Score, mean (SD) scores | 14.7 (5.8) | 15.2 (6.0) | t=-0.774 | 0.439 |
| Glasgow Coma Scale, mean (SD) scores | 14.8 (0.7) | 14.9 (0.5) | t=-0.124 | 0.902 |
| Injury type, N (%) intentional | 70 (8.9) | 11 (10.1) | χ^2^=0.164 | 0.686 |
| Hospital Anxiety Depression scale-anxiety subscale, mean (SD) scores | 2.7 (3.2) | 5.9 (4.8) | t=-6.626 | **<0.001** |
| Hospital Anxiety Depression scale-depression subscale, mean (SD) scores | 5.2 (4.7) | 8.6 (5.2) | t=-6.436 | **<0.001** |
| Heart rate per minute, mean (SD) | 78.7 (11.3) | 79.0 (10.7) | t=-0.280 | 0.779 |

^a^ t-tests or χ^2^ tests, as appropriate.

**Bold style** indicates statistical significance (P-value<0.05).
